# Laterally Orienting *C. elegans* Using Geometry at Microscale for High-Throughput Visual Screens in Neurodegeneration and Neuronal Development Studies

**DOI:** 10.1371/journal.pone.0035037

**Published:** 2012-04-20

**Authors:** Ivan de Carlos Cáceres, Nicholas Valmas, Massimo A. Hilliard, Hang Lu

**Affiliations:** 1 Interdisciplinary Bioengineering Graduate Program, Georgia Institute of Technology, Atlanta, Georgia, United States of America; 2 Queensland Brain Institute, The University of Queensland, Brisbane, Queensland, Australia; 3 School of Chemical & Biomolecular Engineering, Georgia Institute of Technology, Atlanta, Georgia, United States of America; Inserm U869, France

## Abstract

*C. elegans* is an excellent model system for studying neuroscience using genetics because of its relatively simple nervous system, sequenced genome, and the availability of a large number of transgenic and mutant strains. Recently, microfluidic devices have been used for high-throughput genetic screens, replacing traditional methods of manually handling *C. elegans*. However, the orientation of nematodes within microfluidic devices is random and often not conducive to inspection, hindering visual analysis and overall throughput. In addition, while previous studies have utilized methods to bias head and tail orientation, none of the existing techniques allow for orientation along the dorso-ventral body axis. Here, we present the design of a simple and robust method for passively orienting worms into lateral body positions in microfluidic devices to facilitate inspection of morphological features with specific dorso-ventral alignments. Using this technique, we can position animals into lateral orientations with up to 84% efficiency, compared to 21% using existing methods. We isolated six mutants with neuronal development or neurodegenerative defects, showing that our technology can be used for on-chip analysis and high-throughput visual screens.

## Introduction

Knowledge of the molecular mechanisms underlying neurodegeneration and neuronal development are critical for advancing the treatment of neurological pathologies; however, these processes are not fully understood. The nematode *Caenorhabditis elegans* presents an exquisite opportunity to elucidate some of these pathways because of the ease of genetic manipulation and the high level of conserved mechanisms between the nematode and vertebrates. For example, like that of mammals, the development of the *C. elegans* nervous system is regulated by common guidance and polarity cues such as Netrin, Slt, Wnt, and Par protein complexes [1], [2], [3], [4], [5]. Furthermore, *C. elegans* models for neurodegenerative diseases such as Alzheimer's, Parkinson's, and Huntington's are also available, and are being used to elucidate the molecular mechanisms behind these diseases [6].

Unbiased forward genetic screens are commonly used in *C. elegans* to discover genes involved in specific biological processes [7]. This procedure involves searching through large populations of randomly mutagenized individuals to find those that harbor a mutation causing the phenotype of interest. In recent years, researchers have begun to use microfluidics for handling *C. elegans*, in order to facilitate animal manipulation and increase overall throughput of genetic and pharmacological screens [8], [9], [10], [11]. In addition to screening applications, microfluidic methods have significantly reduced the amount of time-consuming manual operations normally required for different *C. elegans* experimentations, such as culture, laser-axotomy, and cell-ablation [12], [13], [14].

For many applications utilizing these microfluidic chips, a specific nematode body orientation is required. For example, visual inspection of neurons and their processes requires animals to be oriented with these structures as close as possible to an imaging objective without visual obstruction by other tissues. Similarly, a specific body orientation facilitates inspections of neuronal features, such as neurite trajectories [15], [16]. The *C. elegans* D-type motor neurons perfectly exemplify this situation. This class of 19 GABAergic neurons is crucial to the normal coordinated locomotion of *C. elegans*, and can be highlighted in animals carrying the *juIs76* transgene, which expresses green fluorescent protein (GFP) under the control of the *unc-25* (a gene encoding glutamic acid decarboxylase) promoter [7]. When visualized with *juIs76*, the D-type motor neurons show discrete single process commissures that run between the ventral and dorsal nerve cords (VNC and DNC, respectively) for the entire animals' body [6]. These commissures provide multiple opportunities to visualize phenotypic defects, when the animal is oriented in a precisely lateral position, and are ideal for studying neurodevelopmental and neurodegeneration abnormalities. While techniques to influence anterior versus posterior entry into microfluidic channels exist [17], no methods have yet been reported to bias lateral orientation.

Here we present a simple microfluidic device designed to passively orient *C. elegans* by exploiting a curved channel geometry. We conducted a pilot forward genetic screen for neurodevelopmental and neurodegeneration phenotypes and isolated six independent mutants, demonstrating that on-chip analysis and high-throughput visual examination can be performed using our design. We also show that our device can be used on animals without altering viability or reproductive capabilities. The advantages of using this system are threefold. First, our curved channel geometry orients animals passively, which makes the operation simple and robust, and facilitates high-throughput analysis of *C. elegans* strains requiring lateral orientation. Second, the curved design increases nematode body area within the microscope field of view, reducing the need to move the sample in order to inspect the entire worm body. Finally, the system is comparatively simple, potentially allowing non-experts to operate the device.

## Results

### Sample loading orientation and device characterization

**Figure 1:**
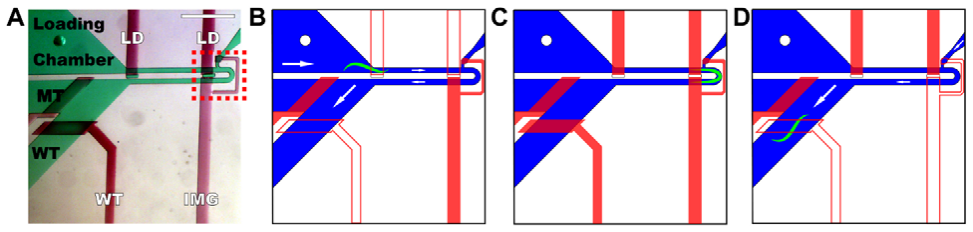
Microfluidic device to passively orient *C. elegans* for visual screening. (A) Device used for orienting, imaging, and sorting animals. Flow layer is shown in green with black text, valve control layer in red with white text. LD is loading valve, MT is channel for mutant output, and WT is channel (black) and valve (white) for wild-type output. IMG is imaging valve. Not labeled are the mutant valve (right of mutant channel) and flush channel (green area above red box). Input and flush channel fluid flow are controlled off-chip. Imaging area is indicated by red dashed box. Scale bar is 800 µm. (B) Device schematic of valve state during worm loading. Worm is driven into imaging area using positive input pressure to induce fluid flow. Loading valve is not actuated, allowing fluid flow. Imaging valve is actuated to prevent worm from exiting imaging area. Wild-type valve is not actuated to assist in worm loading and to provide an exit should an animal slip past the imaging valve. Mutant valve is actuated to prevent animals from entering mutant output. (C) Device schematic of valve state during analysis. All valves are actuated and input pressure is cut off halting fluid flow. (D) Device schematic of valve state during worm sorting. Example for wild-type sorting is shown. Worm is driven out of the imaging area by positive pressure from the flush channel. Loading valve is actuated to prevent any other animals from entering the imaging area. Imaging valve is not actuated to allow worm exit. Wild-type valve is not actuated to allow worm exit into wild-type output. Mutant valve is actuated to prevent animals from entering mutant output. Mutant and wild-type valve state is reversed when mutant sorting is performed. Fluid flow direction indicated by white arrows and is not proportional to size of arrow. Non-filled and filled red boxes indicate non-actuated and actuated valves respectively.

**Figure 2:**
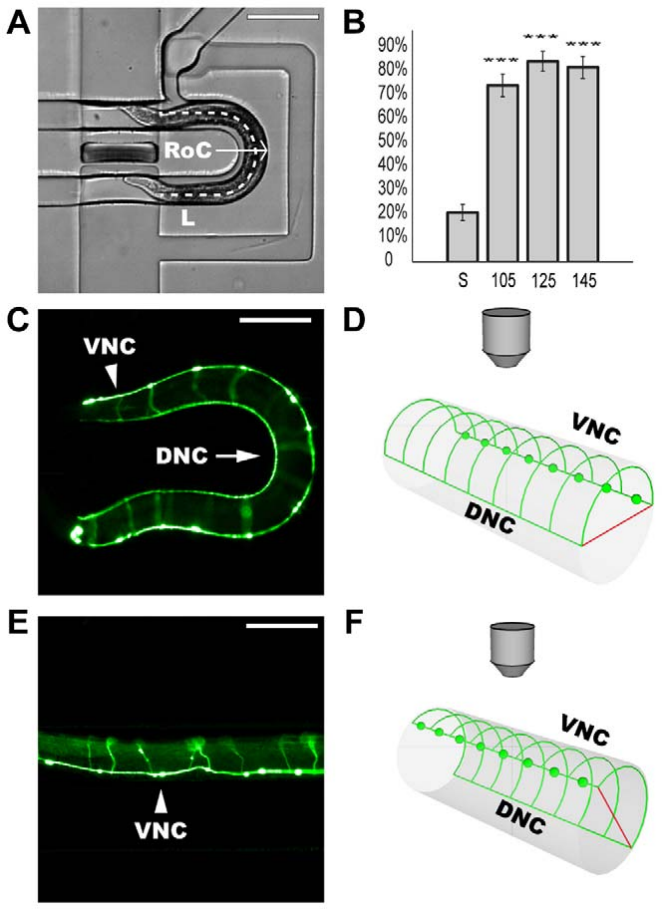
Effect of curved channel on animal orientation. (A) Zoomed in view of imaging area shown by dashed red box from Figure 1A. White arrow indicates device radius of curvature (RoC) from arc center to outer edge. Dashed line indicates length (L) between loading and imaging valves. Scale bar is 200 µm. (B) Frequency of lateral nematode orientation for various channel geometries with standard error of proportion. Triple asterisk indicates statistical significance compared to straight channel designs (*p*<0.001 determined using chi-squared test). S represents straight channel, while remaining labels indicate the 105 µm, 125 µm, and 145 µm RoC devices respectively. L for all devices is 700 µm. (C) Nematode oriented laterally in curved channel device (both nerve cords visible). Commissures present on different focal plane are obscured. (D) 3-D model of animal body section and microscope objective (viewpoint reference) showing nerve cord placement for a laterally oriented animal. (E) Nematode in a non-lateral body orientation as observed when loading animals into straight channel devices; DNC not visible due to animal orientation. Animal is within the same field of view as seen in panel C. Arrowheads for images (C) and (E) indicate ventral nerve cord (VNC) determined by placement of VD and DD motor neuron cell bodies. Arrow indicates dorsal nerve cord (DNC). Scale bars are 100 µm. Transgene marker for all fluorescent images is *juIs76(Punc-25::GFP*). (F) 3-D model of animal body section and microscope objective for non-lateral oriented animal. Model diagrams (D) and (F) not drawn to scale. Red lines illustrate dorso-ventral axis. Green spheres represent DD and VD neuron cell bodies.

The ability to consistently load animals into a specific orientation is often necessary for the visual detection of defects in genetic screens. The lateral body orientation of *C. elegans* is commonly seen in freely moving animals on an agar plate. This orientation is also the most useful for analyzing neuronal processes that travel along the antero-posterior axis, as well as processes that travel laterally across the worm body. In this work, we show that *C. elegans* preferentially adjust themselves into this lateral orientation as a result of the curved geometry of our device. We designed a microfluidic chip containing a novel curved microchannel, with a radius of curvature (RoC) of 125 µm, which laterally oriented animals with an efficiency of 84±4% (mean ± standard error, *n* = 76) (Figure 1, 2A–D). Comparatively, animals within straight channel devices, of similar channel width, orient laterally only 21±3% of instances (*n* = 145) and were otherwise rotated along the antero-posterior axis (Figure 2E,F). Decreasing or increasing the RoC of the curved microchannel by 20 µm did not have a statistically significant effect on orientation efficiency (*p*>0.4, chi-squared test), resulting in laterally oriented animals with frequencies of 74±5% (*n* = 84) and 82±5% (*n* = 71), respectively. Locomotory impaired animals (*unc-71*) similarly displayed a high efficiency of lateral orientation (68±5%, *n* = 75) within the device; in addition, animals with gross morphological defects, such as long (*lon-3*) or dumpy (*dpy-4*) mutants, also oriented laterally at a higher efficiency (72±5%, *n* = 75 for *lon-3* and 57±6%, *n* = 76 for *dpy-4*). Therefore, we conclude that the gross morphology of animals will not considerably impact the orientation efficiency of our device, making it suitable for operation with mutagenized populations.

To further characterize loading orientation bias in our curved channel device, we measured whether the worm's ventral or dorsal side faced the inside curve of the imaging area, along with whether the worm entered the imaging area head or tail first. We found that our curved device favors head entry of animals into the imaging area in 70±6% of instances compared to 30±6% for tail entry, (mean ± standard error, *n* = 69, *p*<0.01, chi-squared test). Additionally, the device also had a ventral bias of 59±6% versus a dorsal bias of 41±6% (*n* = 69, *p*>0.1). When combined with our lateral orientation method, head-to-tail entry bias provides an opportunity to preferentially load animals into the device in known orientations, facilitating image acquisition and analysis of specific locations of the animal's body.

Our experiments suggest that curved channel geometries passively orient *C. elegans* with greater than 80% efficiency, while orientation within straight channel devices is random. Curved channels are therefore the best geometry to employ for examining *C. elegans* features aligned in the dorso-ventral plane, or along the lateral positions of the animals' body.

To verify compatibility of using our novel device for high-throughput animal handling and to estimate sorting efficiency, we performed a mock screen including animals expressing GFP in different neurons (mechanosensory neurons, *zdIs5* transgene) and with an elongated phenotype (*lon-3*). We successfully recovered 100% of *lon-3* (*zdIs5*) mutants (*n* = 10) from a population of ∼1,000 wild-type (*juIs76*) animals with no false positives (i.e. no *juIs76* animals were sorted as *lon-3; zdIs5* animals). In addition, we also assessed whether on-chip manipulation altered animal viability or egg-laying in wild-type worms 48 hours after chip operations. All animals survived after being sorted through the microfluidic device, and the total number of eggs laid per animal in the manipulated population, 195±6 (mean ± standard error, *n* = 15), had no statistical difference when compared to a control population, 188±8 (*n* = 14, *p*>0.5, Student's *t*-test). Thus, results of the device characterization experiments validate our method to orient animals within a microfluidic chip, and confirm its efficacy for high-throughput applications while having no adverse effects on animal viability or reproduction.

### Identification of neurodegenerative and neurodevelopmental mutants using curved channel microfluidic devices

**Figure 3:**
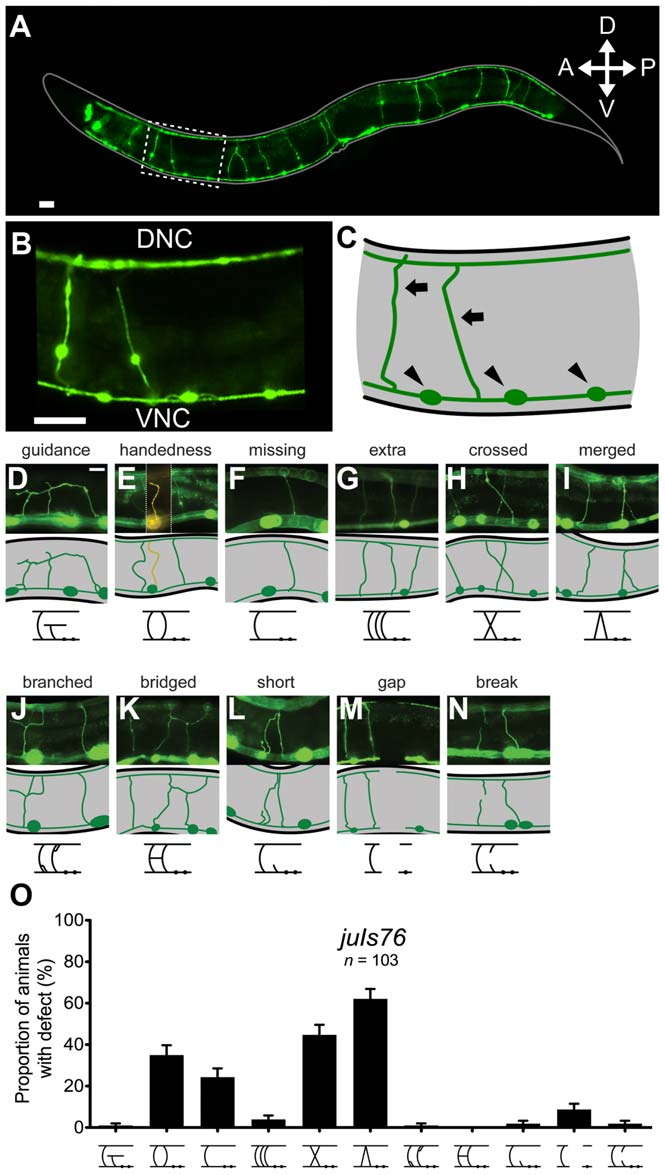
Morphology of wild-type and defective D-type motor neuron commissures. (A) Full body image of adult worm. Scale bar is 25 µm. (B) Expanded view of area within dashed box in panel A. Ventral nerve cord (VNC) is distinguished by presence of neuronal cell bodies. Dorsal nerve cord labeled DNC. Scale bar is 25 µm. (C) Representative image of panel B. Black arrows label neuronal commissures while black arrowheads identify neuronal cell bodies. Respective phenotypes characterized by: (D) commissure never reaching dorsal nerve cord; (E) commissure running along the opposite side of the animal's body (left, colored yellow); (F) absent commissure; (G) additional commissure present; (H) two commissures crossing over each other; (I) two commissures entering the dorsal nerve cord or leaving the ventral nerve cord together, they may also partially fasciculate; (J) bifurcating commissure; (K) neighboring commissures joined by a process; (L) neurite with length less than half nematode width; (M) an absence of GFP expression along either dorsal or ventral nerve cords; (N) break in GFP expression in a commissure. Scale bar is 25 µm. Representative image of phenotype shown beneath each photo accompanied with illustrative phenotype symbol. (O) Proportion of animals in a population with at least one incidence of each independent defect. Error bars represent standard error of proportion.

To demonstrate the utility of this simple and easy-to-use methodology, we performed a pilot screen to isolate novel mutants with neurodegenerative and neurodevelopment phenotypes in the D-type motor neurons. We analyzed approximately 10,000 F2 progeny on-chip (∼1000 haploid genomes), at a consistent rate of 600±45 animals per hour (mean ± standard error). Throughput was calculated from two separate but identical devices over the course of four days of screening. In wild-type animals, 17 out of 19 of the D-type neurons, which belong to either the DD class (six cells) or the VD class (13 cells), send commissures along the right side of the animal's body (Figures 3A–C) [18]. Therefore, strains expressing the *juIs76* transgene are best inspected when the right sides of animals are closest to the objective, with their dorso-ventral plane parallel to the cover-glass. Examining DD or VD commissures located on the opposite (left) side of the animal body is confounded due to optical artifacts (e.g. scattering by the animal's body tissue). Therefore, commissures were only evaluated for animals loaded into the device with the correct body orientation (right side of body closest to microscope objective). The DNC and VNC were evaluated in all animals positioned laterally, since both nerve cords are visible in this orientation. From this screen, we successfully isolated six independent mutant alleles (*a070*, *a071*, *a073*, *a074*, *a077*, and *vd029*), which present abnormalities in their GFP-labeled neuronal processes and commissures.

### Phenotype characterization of mutant animals

**Figure 4:**
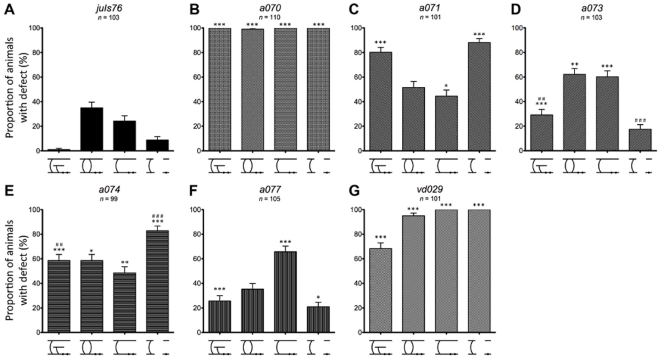
Adult phenotype characterization. (A–G) Proportion of animals in a population with at least one incidence of each of the independent defects: guidance, handedness, missing, and gap for wild-type (*juIs76*) and newly isolated mutants. Asterisks indicate statistical significance of phenotype compared to wild-type. Single, double, and triple asterisks indicate *p*<0.05, *p*<0.01, and *p*<0.001, respectively. Pound signs indicate statistical significance of phenotype compared between *a073* and *a074*. Double and triple pound signs indicate *p*<0.01 and *p*<0.001, respectively. Statistical significance determined using chi-squared test. Number of animals examined for each allele labeled in graph. Error bars represent standard error of proportion.

Following isolation, we further characterized the phenotypic defects of the mutant animals in relation to the commissures of the DD and VD motor neurons. Inspections of these neurons lead to the classification of defects into eleven categories (shown and described in Figures 3D–N), of which the main ones included misguided commissures (guidance), commissures travelling on the wrong side of the body (handedness), lack of commissure (missing), and visible interruptions in the nerve cords (gaps). The frequency of these phenotypes in animals of the wild-type *juIs76* strain is shown in Figure 3O, and data for our isolated mutants is shown in Figures 4 and S1. Phenotypes were scored independently of each other, as most mutant animals presented more than one type of defect.

**Figure 5:**
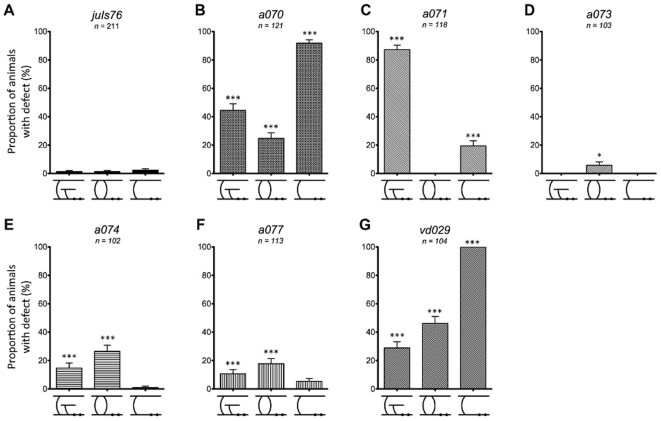
L1 phenotype characterization. (A–G) Proportion of animals in a population with at least one incidence of each of the independent defects: guidance, handedness, and missing defects seen for wild-type (*juIs76*) and newly isolated mutants. Asterisks indicate statistical significance of phenotype compared to wild-type. Single, double, and triple asterisks indicate *p*<0.05, *p*<0.01, and *p*<0.001 respectively. Statistical significance determined using chi-squared test. Number of animals examined for each allele labeled in graph. Error bars represent standard error of proportion.

**Figure 6:**
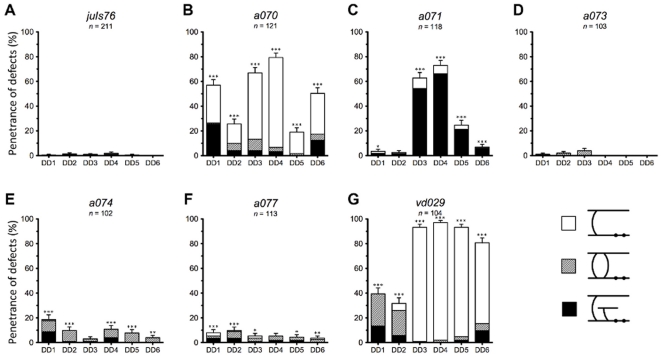
Cell specific characterization for L1 animals. (A–G) Penetrance of defects in each DD neuron for wild-type (*juIs76*) and isolated alleles. Asterisks indicate statistical significance of combined phenotypes per cell compared to wild-type. Single, double, and triple asterisks indicate *p*<0.05, *p*<0.01, and *p*<0.001, respectively. Statistical significance determined using chi-squared test. Number of animals examined for each allele labeled in graph. Error bars represent standard error of proportion for sum of phenotypes per cell.

To further characterize the mutants isolated during our visual screen, we investigated the developmental onset and cell-specificity of the observed morphology defects. At the L1 juvenile stage, only the six neurons of the DD class are present, as the remaining 13 VD neurons have not yet developed. This allows the precise identity of each defective DD neuron to be determined, and presents an opportunity to inspect whether the embryonically and post-embryonically developing cells are differently affected. The frequency with which defects occurred in populations of young L1 stage animals is shown in Figures 5 and S2, and is assembled by individual cell identity in Figures 6 and S3.

*a070* and *vd029* are the most defective mutants, with a high occurrence of most of the phenotype scored (Figures 4B, 4G, and S1). Analysis of the six DD neurons at the L1 stage (Figures 5B, 5G and S2) showed that in *a070* the left-right asymmetry (handedness) defect was strongly reduced (about 20%), indicating that the mutation mostly affected the VD neurons. On the contrary, the missing commissure defect for *a070* was already fully penetrant at this stage (90%), indicating that it affected all the D-type neurons, with the DD3 and DD4 neurons being particularly susceptible (Figures 6B and S3). In *vd029*, there was a strong effect on the DD class, with these animals lacking the DD3–DD6 neurons (Figures 5G and 6G), and DD1 and DD2 displaying both handedness and guidance defects.

In *a071* and *a074*, over 80% of the animals presented gaps in GFP expression on either the ventral or dorsal nerve cords (Figures 4C and 4E). While this phenotype correlated with a strong guidance defect (80%) in *a071*, in *a074* the guidance defect was less penetrant. Interestingly, the guidance defect of *a071* was also highly penetrant in the L1 stage with the DD3–DD6 cells presenting this defect (Figures 5C and 6C). On the contrary, L1 animals of the *a074* strain had very minor defects (Figure 5E, and 6E), indicating a much later onset and/or selective involvement of the VD neurons.

*a077* and *a071* were isolated from the same pooled population of F2s; however, complementation analysis indicated they are not alleles of the same gene (Figure S1). Characterization of these two mutants indicated that while guidance and gap defects were both highly penetrant in *a071*, they were only minor in *a077*, which presented a high penetrance of missing commissures instead (Figures 4C and 4F). Even more strikingly, at the L1 stage, the guidance defects in *a071* were at around 85% while only 10% in *a077* (Figures 5C, 5F, 6C and 6F).

Lastly, *a073* presented handedness and missing commissures phenotypes (Figure 4D), mostly caused by defects in the VD neurons as both these phenotypes were highly reduced in the L1 stage (Figure 5D, 6D).

To confirm that our isolated mutants bred true, we outcrossed each of them with wild-type male animals carrying the *zdIs5(Pmec-4::GFP)* transgene as a marker for crossed progeny. We were able to recover the original phenotypes in the F2 generation for all of our strains (Figure S1). Additionally, by examining the heterozygous F1 generation we determined that all of our mutations are recessive, with the exception of *a077*, which has a semi dominant effect (Figure S1).

## Discussion

We present a microfluidic design capable of orienting *C. elegans* on-chip by using device geometry to position the nematode body into lateral orientations. This alignment mimics the lateral orientations normally seen during standard analysis of *C. elegans* on agarose pads. The design is comparatively simple to fabricate, requires no extra systems other than pressure sources to operate valves, and can be easily altered to compensate for different body sizes and morphologies if the starting population requires so. We demonstrated the utility and advantages of our technology compared to standard straight channel designs with a high-throughput pilot screen by successfully isolating mutants involved in neuronal development and degeneration. This technology is beneficial to visual inspections of features along the antero-posterior or dorso-ventral body axes. This includes processes and commissures similar to those of the VD and DD neurons, such as in the DA, DB, and AS motor neurons, neuromuscular synapses along both the ventral and dorsal nerve cord, mechanosensory neurons, and other dorso-ventrally aligned body tissues [15]. Our device will thus extend the tools available in *C. elegans* to perform screens to identify genes involved in numerous phenotypes of interest.

The DD and VD motor neurons are ideal cells with which to measure the efficacy of orientation techniques, as their morphology is optimally displayed in laterally oriented worms (Figure 2C), which have commissures curving toward the imaging objective (Figures 2D and 2F). In our curved channel design, GFP expressed in both the ventral and dorsal nerve cords can be seen along the curvature of the microchannel, indicating that the animal is in a lateral orientation, with the dorso-ventral plane parallel to the cover glass. The advantage of this orientation is that it allows for inspection of both the VNC and DNC, in addition to the DD and VD commissures, along the entire length of the animal, presenting multiple opportunities to observe defects in neuronal morphology. In contrast, GFP expression of morphological features of animals viewed in a straight channel device can be out of focus and obscured (Figure 2E and 2F), resulting in concealed portions of connecting commissures, which ultimately makes phenotypic analysis time-consuming and difficult. Another advantage of the curved channel is that the design allows an animal's entire body to be inspected within a single field of view, while a straight channel design would require multiple fields of view for proper inspection (Figures 2C and 2E).

Analysis of animals during our pilot screen was performed by visually inspecting them on-chip for defects in neuronal morphology. During our screens, we estimate that device operation cycles at about seven seconds, taking into account the entire process of loading, inspecting, and sorting each individual animal. Typically, about two seconds are spent loading each animal into the imaging area, followed by a visual inspection period of up to five seconds. Once the phenotype is determined, the animal is sorted to its appropriate output in less than one second. Taken together, our estimated rate of operation of seven seconds per animal corresponds to a repeatable and measured throughput of over 500 animals per hour.

We isolated over 50 mutants in our pilot screen; however, strains that were sterile or less than 30% penetrant for defects when re-examined were discarded. Conversely, some mutants of interest may have been missed during our screen because they were not fully penetrant. Additionally, we may have discriminated against slow-growing mutants because the growth rate of mutagenized animals varies and we did not include all mutagenized worms in this screen. These artifacts, however, are also present in traditional screens and can be avoided by isolating the F1's and expanding the screened pool of animals [19].

Characterization of our newly isolated mutant strains revealed correlations between individual phenotypes, which may be indicative of specific biological significance. For example, the low penetrance of GFP interruptions in the dorsal or ventral cords (gap defects) in both *a073* and *a077* (Figures 4D and 4F) suggests that these gaps are not strongly associated with either handedness or missing commissural defects. Furthermore, a comparison of strains *a073* and *a074* (Figures 4D and 4E) show that while similar levels of both handedness and missing commissures defects are seen (*p*>0.7 and *p*>0.2 for each defect, respectively), the penetrance of gaps in GFP expression along the nerve cords between the two alleles are significantly different (*p*<0.001). Interestingly, the difference in penetrance of commissural guidance defects between these strains is also significant (*p*<0.01), suggesting a possible association between highly penetrant commissural guidance defects and the gap phenotype seen in the nerve cords. Alleles *a070*, *a071*, *a074*, and *vd029* similarly display high penetrance of both commissural guidance defects and nerve cord gaps, reinforcing this association.

The phenotypic analysis of L1s allows differentiation between developmental and degeneration phenotypes. For example, alleles *a073*, *a074*, and *a077* demonstrate much lower penetrance of defects when analyzed as L1s than they do as adults. This suggests that while these mutations affect embryonic development, they have an enhanced phenotype at later stages, either due to additional defects that develop post-embryonically or because of aging effects. Secondly, cell specific data reveals the susceptibility of specific body sections to both guidance and missing commissure defects. The majority of highly penetrant defects present for our isolated alleles are concentrated in the animal mid-body or posterior (neurons DD3–DD6), as observed in strains *a070*, *a071*, and *vd029*. This may be indicative of a higher susceptibility to developmental or maintenance defects of these cells or of this entire region of the body.

Like all microfluidic techniques, certain considerations must be taken into account when using this device for specific applications. For example, while our design passively orients animals into lateral positions, our system cannot bias the position of the ventral or dorsal side. Another key point is recognizing that device throughput is ultimately dependent on the user's familiarity with, and the overall complexity of, the phenotype analysis, and to a certain extent user's familiarity with microfluidic devices. Additionally, while we tested animals with body morphology defects using slightly modified designs, extreme body shapes may present complications in device operation (i.e. problems in loading, orientating, and sorting). The advantage of our microfluidic device, however, is in streamlining screening preparation, and worm-handling as it does not involve mounting animals on slides, waiting for anesthetics to take effect, transporting animals to a microscope for imaging, and careful rescue of the animals from slides, as conventional methods require. Comparatively, our device allows for a single manipulation to load, image, and sort the relevant mutants, requiring only an initial setup time of 20 to 30 minutes before screening.

Results presented here suggest that our microfluidic screening method allows discovery of mutants of interest by exploiting high-throughput techniques to examine large numbers of nematodes in pooled populations. We expect this type of design to be useful in other developmental and functional screens where animals are to be routed and imaged in particular orientations.

## Materials and Methods

### Microfluidic device fabrication and operation

We designed and fabricated a two-layer microfluidic device using polydimethylsiloxane (PDMS, Dow-Corning) and standard multi-layer soft lithography techniques [20]. A loading chamber was designed to store nematodes in the device until they are sent to the imaging area (red dashed box in Figure 1A) to be analyzed and subsequently sorted through one of two exits. Nematode loading and sorting within the device was controlled through the actuation and use of partially-closed valves in conjunction with pressure-driven flow (Figures 1B, 1C, and 1D). To reduce imaging chamber distortion during analysis, control channels were filled with a 58% glycerol solution [21].

Device geometry was designed by selecting values similar to worm body dimensions. Channels within the imaging area are about the size of a gravid adult, 70 µm in width, while the standard arc length (L) between loading and imaging valves is 700 µm to help restrict animal movement. The arc length between the loading and imaging valves was modified to approximately 500 µm or 900 µm to accommodate for *dpy-4* and *lon-3* mutants whenever necessary. Standard radius of curvature (RoC) for the curve in the imaging area is 125 µm from the arc center to the outer edge of the channel to allow for a 20 µm increase or decrease, while still meeting mask printing space requirements. Lastly, flow channel height is approximately 75 µm, which is also slightly larger than the diameter of an adult nematode.

### Orientation analysis

*C. elegans* were analyzed in various microfluidic designs to test locomotory defects, body type differences, and the effect of the imaging area's RoC on lateral orientation. Animals were grown until gravid adults and then evaluated. Five-second videos were captured per animal and were visually analyzed to determine animal orientation.

For wild-type animals, videos were acquired at 32 frames per second using a CCD camera (Hamamatsu C9100-13), and were recorded using a 20×/0.5NA magnification objective in fluorescence mode on a wide field upright compound microscope (Leica DM4500). Videos for *dpy-4 juIs76* and *lon-3 zdIs5* mutants were acquired at 26.1 frames per second using a CCD camera (Lumenera INFINITY3 1M) and were recorded using a 20×/0.4NA, and a 10×/0.25NA magnification objective respectively. Videos for these mutants were also recorded in fluorescence mode on a wide field inverted compound microscope (Leica DMI6000 B).

Animals carrying the *juIs76* transgene were scored as laterally oriented if GFP-labeled dorsal and ventral nerve cords could be clearly seen throughout the duration of the video. Conversely, animals were scored as not lateral if either nerve cord was obstructed at any point along the worm body due to a rotated body position within the microchannel. In animals carrying *zdIs5* transgene, since both ALM and PLM are bilaterally symmetric neurons, animals were scored as laterally oriented if only one neuronal process (either left or right) could be clearly seen throughout the duration of the video for the anterior and posterior body (ALM and PLM respectively). Conversely, if both the left and right processes for either ALM or PLM were seen in the same focal plane at any time during the video, animals were scored as not lateral. Expression of GFP in first and last quarters of the animal's body (head and tail) was ignored since the tapered nature of the worm makes both ALML/ALMR and PLML/PLMR processes visible in the same focal plane.

### *C. elegans* culture, mutagenesis, and phenotype scoring

*C. elegans* strains used in these studies were *juIs76(Punc-25::GFP)*, *zdIs5(Pmec-4::GFP)*, CZ1931 *unc-71(ju156) juIs76(Punc-25::GFP)*, QH3736 *lon-3(e2175) zdIs5(Pmec-4::GFP)*, QH3833 *dpy-4(e1166) juIs76(Punc-25::GFP)*, and CX8600 *kyIs417(Podr-1::dsRed, Pgcy-36::GFP)*. All animals were grown between 20°C and 25°C using established culturing protocols [18], [22]. Mutagenesis was performed using standard techniques and concentrations of the chemical mutagen ethyl methanesulfonate (Sigma Aldrich) to perform a pooled F2 screen on wild-type *juIs76* animals [23].

F2 progeny were cultured for 2.5 to 3.5 days (dependent on cultivation temperature) and visually examined on-chip. Animals were isolated if they presented, any breaks or gaps in neuronal processes, misguided commissures, or any gross difference in neural morphology when compared to wild-type. Animals isolated for exhibiting phenotypic abnormalities were further examined on agarose pads using 5 mM sodium azide (Sigma Aldrich) or 0.01% tetramisole (Sigma Aldrich) as an anesthetic [24].

Adult animals three to four days old were analyzed for morphological defects in each commissure of 16 of the 19 D-type motor neurons. The VD1 neuron was omitted as its morphology is confounded by the RME neurons; in addition, the DD1 and VD2 commissures could not be accurately scored as they travel on the opposite side of the body (this was not the case in the *vd029* mutant, so all these cells were also scored). Larvae were analyzed early in the L1 stage, within six hours of hatching and prior to the development of the VD neurons, so that all six DD neurons could be individually scored.

Animals were visualized with 20×/0.5NA, and 40×/0.75NA magnification objectives on a wide field upright compound microscope (Zeiss Axio Imager Z1). Images were captured using a CCD camera (Photometrics CoolSNAP HQ^2^), and Z-stacks were manually flattened to a single plane in Adobe® Photoshop® CS3.

### *C. elegans* mock screen and egg-laying assay

For the mock screen, M9 solution containing approximately 1,000 wild-type (*juIs76*) adult animals was prepared. Ten adult *lon-3* (*zdIs5*) mutants were then individually picked and placed into the solution. This population of animals was then sorted through our microfluidic device and then inspected under a dissecting microscope using high magnification to verify the phenotype of recovered animals. All animals were grown at 20°C.

To compare egg laying rates, animals were grown at 20°C until young adults and then separated into two populations. One population was then sorted through our microfluidic device. After 48 hours, progeny of the two populations were manually counted under a dissecting microscope at low magnification.

## Supporting Information

Figure S1 Proportion of adult animals in a population with at least one incidence of each independent defect (%).
(TIFF)

Figure S2 Proportion of L1 animals in a population with at least one incidence of each independent defect (%).
(TIF)

Figure S3 Penetrance of defects per cell in L1 populations with at least one incidence of each independent defect (%).
(TIF)
