# Supplementary figures and images for: Laterally Orienting C. elegans Using Geometry at Microscale for High-Throughput Visual Screens in Neurodegeneration and Neuronal Development Studies
Source: PLoS One. 2012 Apr 20;7(4):e35037. doi: 10.1371/journal.pone.0035037 (PMC3335040; doi:10.1371/journal.pone.0035037)

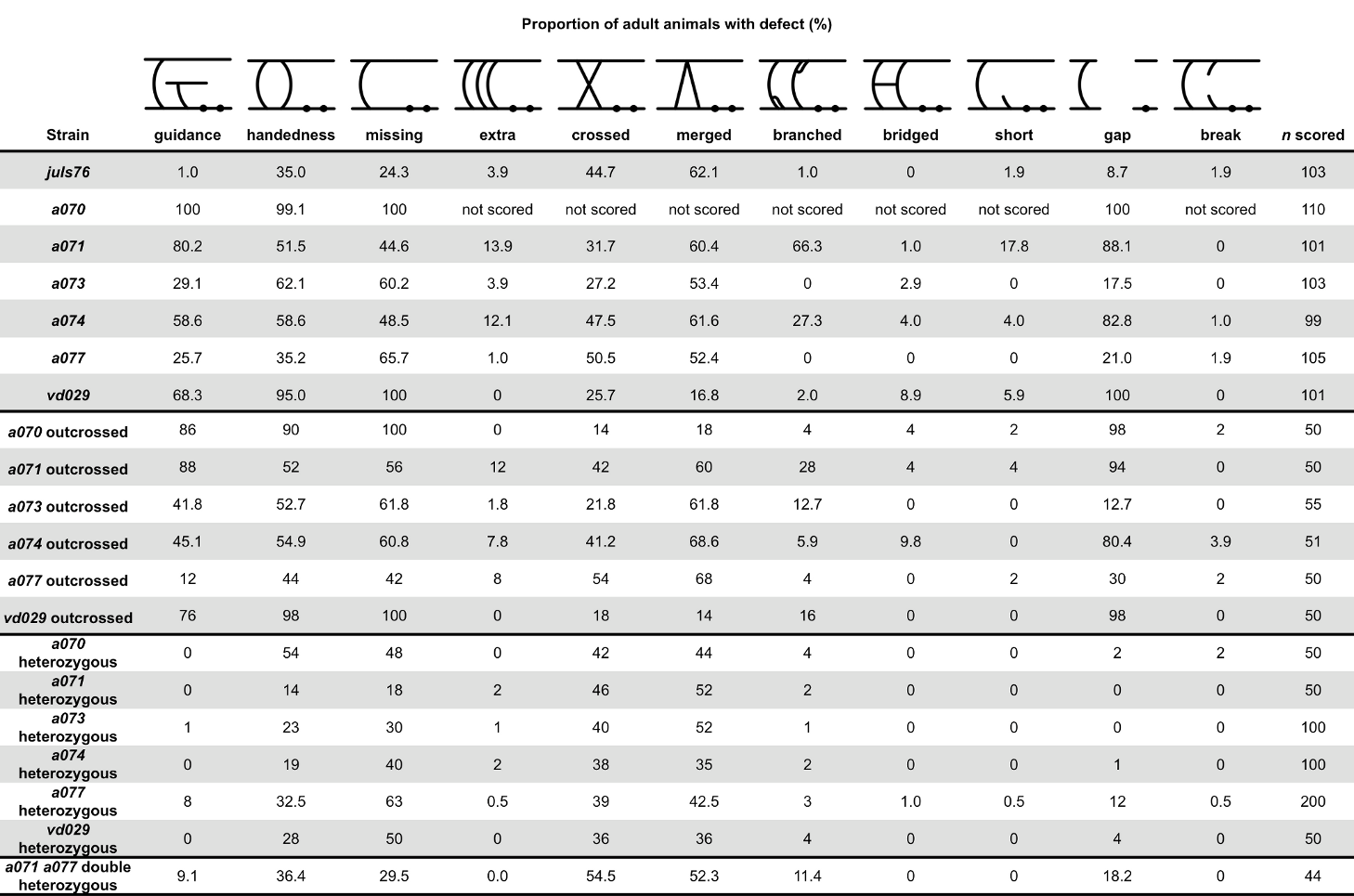

Supplement: Figure S1 — Proportion of adult animals in a population with at least one incidence of each independent defect (%). (TIFF) [file pone.0035037.s001.tif]

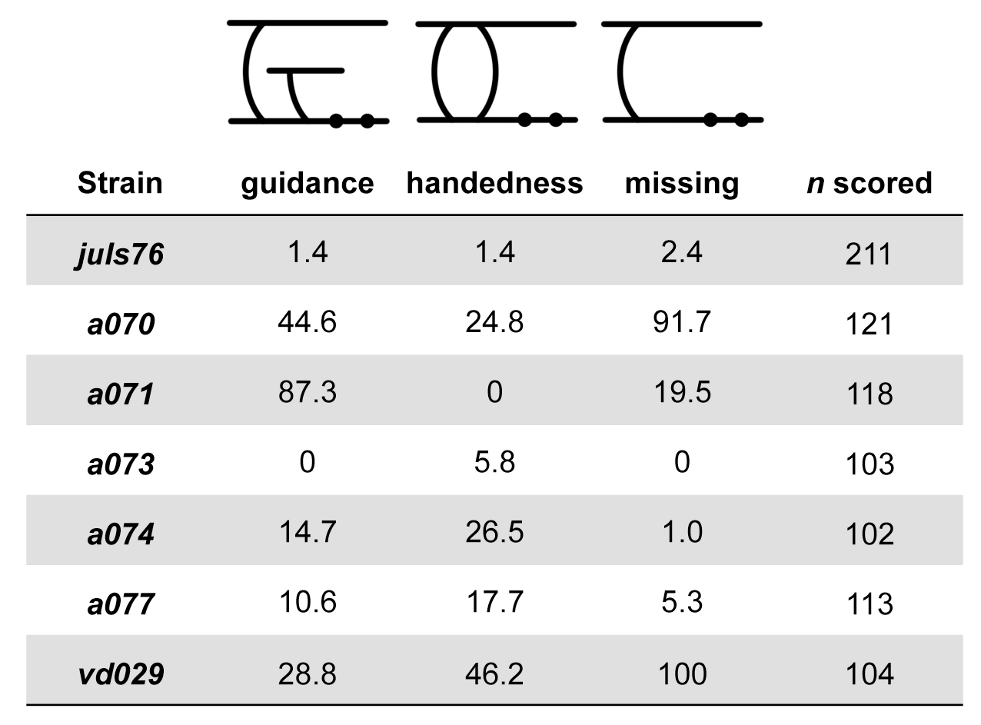

Supplement: Figure S2 — Proportion of L1 animals in a population with at least one incidence of each independent defect (%). (TIF) [file pone.0035037.s002.tif]

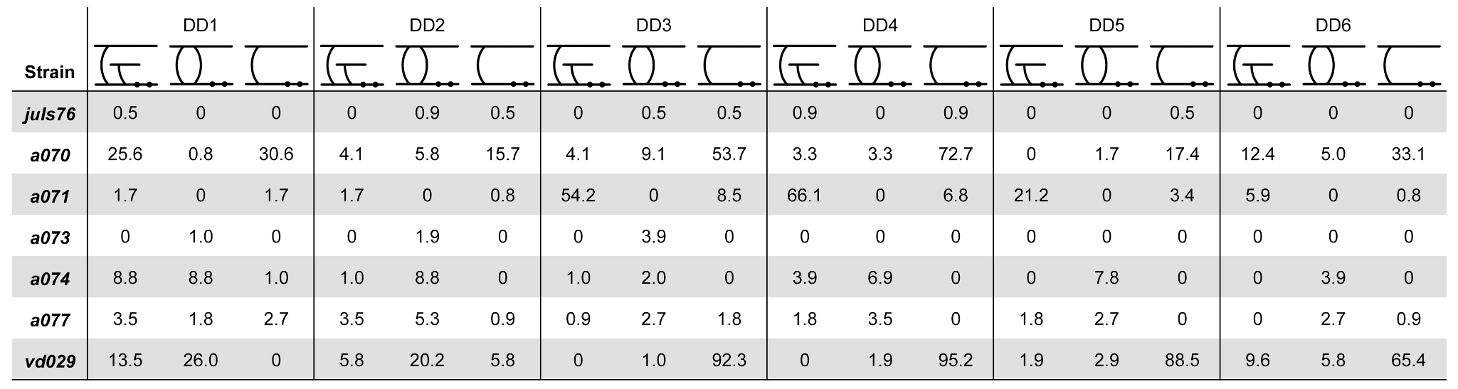

Supplement: Figure S3 — Penetrance of defects per cell in L1 populations with at least one incidence of each independent defect (%). (TIF) [file pone.0035037.s003.tif]
